# CT scan and ultrasound comparative assessment of PEEP-induced lung aeration changes in ARDS

**DOI:** 10.1186/cc13475

**Published:** 2014-03-17

**Authors:** I Algieri, S Mongodi, D Chiumello, F Mojoli, M Cressoni, G Via, S Luoni, A Colombo, G Babini, A Braschi

**Affiliations:** 1 Universitá degliStudi di Milano, Milan, Italy; 2 Fondazione IRCCS Policlinico S. Matteo Hospital - University of Pavia, Italy; 3 Fondazione IRCCS Cá Granda - Ospedale Maggiore Policlinico, Milan, Italy

## Introduction

CT-scan quantitative analysis (qCT) represents the gold standard to assess lung aeration and recruitment in ARDS patients. Lung ultrasound (LUS) has been proposed as a bedside nonirradiating alternative to assess lung recruitability, identifying patients who may benefit from higher PEEP levels. We compared the two methods in the assessment of PEEP-induced lung aeration changes.

## Methods

LUS and whole-lung CT scan were performed on ARDS sedated, paralyzed, mechanically ventilated patients at PEEP 5 and 15 cmH_2_O. LUS was performed considering six areas for each lung, with a comprehensive scan of the intercostal spaces in each area. We assigned to each area a score of aeration [1]: 0 (normal lung), 1 (≥3 noncoalescent B-lines), 2 (≥3 coalescent B-lines), 3 (consolidation). A cumulative LUS score (LUSS, ranging from 0 to 36 for the two lungs) was obtained as sum of all areas' individual scores, each area's score being the average of all pertaining LUS findings. LUS recruiters upon PEEP increase from 5 to 15 cmH_2_O were defined by the switch of at least three areas to well aerated (area score 0). LUS-based assessment of lung aeration and lung recruitability was compared with qCT findings.

## Results

ResultsWe enrolled seven patients (six males, age 54.1 ± 22.2 years, BMI 24.2 ± 4.9 kg/m^2^, PaO_2_/FiO_2 _186 ± 78, tidal volume 445 ± 140 ml, RR 14.5 ± 3.4 breaths/minute, PEEP 12.5 ± 3.3 cmH_2_O). In the 14 conditions evaluated, median LUSS was 19 (IQR 14 to 23); LUSS ≥19 (*n *= 8) corresponded to 34 ± 13% of nonaerated tissue at qCT; LUSS >20 (*n *= 6) to 48 ± 18% (*P *< 0.05). A good linear correlation was found between reduction at LUS of consolidated areas (area score 3) versus reduction of qCT nonaerated volume (*R*^2 ^= 0.66), and between reduction at LUS of poorly aerated areas (area score 1 to 2) versus reduction of qCT poorly aerated volume (*R*^2 ^= 0.74). Change at LUS of at least three areas to well aerated (LUS recruiters, *n *= 4) corresponded to a qCT increase in well-aerated lung volume of 788 ± 262 g versus 431 ± 35 g in the LUS nonrecruiter group (*n *= 3) (*P *< 0.05).

## Conclusion

These preliminary data suggest that LUS could be an accurate tool to assess lung aeration and recruitment at the bedside, avoiding the risks and workload related to the use of CT scan.
